# Home‐Based Management of a Child With Persistent Pulmonary Air Leak Complicated by Pneumonia

**DOI:** 10.1155/crpu/8419647

**Published:** 2026-05-13

**Authors:** Dhruba Shrestha, Sampurna Man Tuladhar, Manoj Krishna Shrestha

**Affiliations:** ^1^ Department of Pediatrics, Siddhi Memorial Hospital (for Women and Children), Bhaktapur, Nepal; ^2^ Department of CTVS, Civil Service Hospital, Kathmandu, Nepal, civilservicehospital.org

**Keywords:** empyema, home-based care, pediatric, pneumonia, pulmonary air leak

## Abstract

**Background:**

Pneumothorax is a condition where air leaks into the space between the visceral and parietal pleura. Community acquired pneumonia is a rare cause of pneumothorax in children. Pneumothorax can sometimes develop as a complication of pneumonia when infection erodes the distal airway into the pleural cavity causing air leak. If the air leak persists for more than 7 days, then it is termed a persistent air leak.

**Case Summary:**

A 6‐year‐old South Asian girl presented with right upper and middle lobe pneumonia with empyema, for which a chest tube drain was inserted, which progressed to a persistent pulmonary air leak. Despite initial hospital management, the air leak persisted, necessitating home‐based care with meticulous follow‐up. Later on, after home‐based care, the child improved and the chest tube could be removed without complications. The management of persistent air leak complicated by pneumonia necessitates a multidisciplinary approach.

**Conclusion:**

This case highlights an innovative approach to managing complex pediatric respiratory conditions outside the hospital setting. The incorporation of domiciliary care introduces a novel dimension. This requires meticulous training of the parents with no medical exposure before monitoring and guiding them via online video and ensuring they adhere to treatment protocols. This report highlights the possibility of conservative domiciliary management for persistent air leak before going into definitive surgery.

## 1. Introduction

Pneumothorax is a condition where air leaks into the space between the visceral and parietal pleura, which causes the lung to collapse [1, 2]. It can be classified into primary or spontaneous pneumothorax, in which the exact cause cannot be identified and secondary pneumothorax, where the inciting cause can be identified for the causation of pneumothorax. Community acquired pneumonia is a rare cause of pneumothorax in children. One of the major causes of secondary pneumothorax is necrotizing pneumonia [3]. Pneumothorax can sometimes develop as a complication of pneumonia when infection erodes the distal airway into the pleural cavity causing air leak. If the air leak persists for more than 7 days, then it is termed a persistent air leak [4].

Air leaks can be of two types: (1) alveolopleural fistula and (2) bronchopleural fistula [5, 6]. Furthermore, air leaks can be of different grades: Grade I: during forced expiration, Grade II: during expiration only, Grade III: during inspiration only, and Grade IV: continuous. Management protocol varies according to the type and severity of air leak [4].

Pneumonia can lead to the development of parapneumonic effusion which subsequently progresses to empyema if it is not managed properly. Empyema can be classified into three distinct stages: (1) exudative stage: early stage after the development of pneumonia, (2) fibropurulent stage: when there is an influx of WBC and bacteria into the pleural cavity after 1–2 weeks, and (3) empyema: fibrin deposition and fluid collection become organized with septations and pus formation [7]. Pneumothorax can develop as a sequelae while draining empyema due to trauma to the visceral pleura and lung parenchyma during the procedure [1]. Management of pneumothorax can become challenging when the air leak is persistent despite medical management.

Here, we present a case of a child who developed a persistent air leak after chest tube drainage of empyema thoracis.

## 2. Case Report

**Figure 1:**
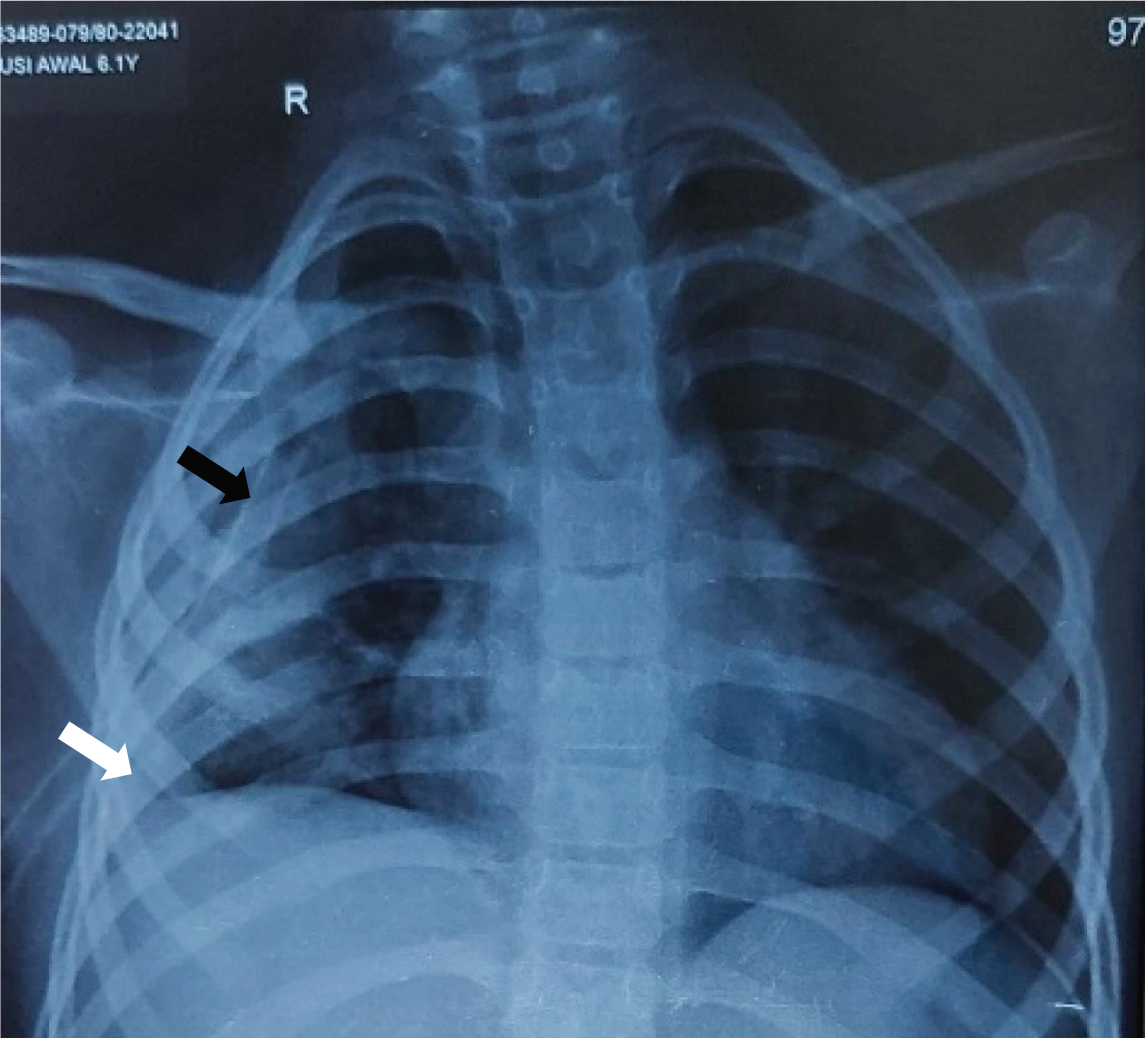
Chest x‐ray showing right pneumonia with pleural effusion (blunted CP angle, white arrow) with a chest tube drain in situ (black arrow).

A 6‐year‐old fully immunized South Asian girl presented with a fever of 6 days, aggravated with 1 day of cough and shortness of breath. She was admitted from ER to the hospital because of right upper and middle zone consolidation detected in chest x‐ray (Figure 1), decreased air entry on the right side, bilateral crepitations, elevated CRP of 48, and oxygen saturation not maintained in room air consistent with a diagnosis of pneumonia. Her oxygen saturation was maintained up to 95% on 2 L of oxygen via nasal prongs. Treatment was started with intravenous Cefotaxime.

**Figure 2:**
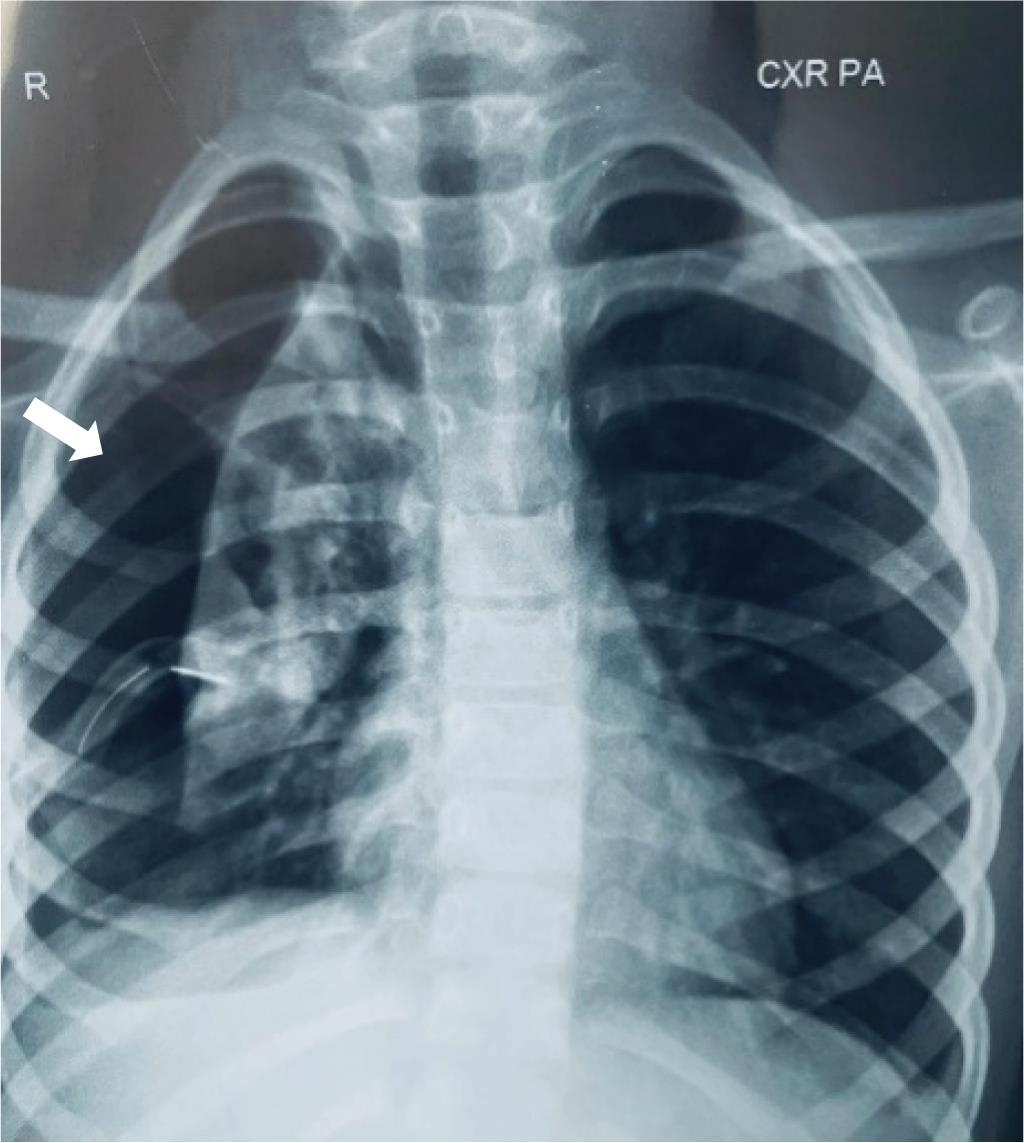
Right‐sided pneumothorax (white arrow) with chest tube in situ.

**Figure 3:**
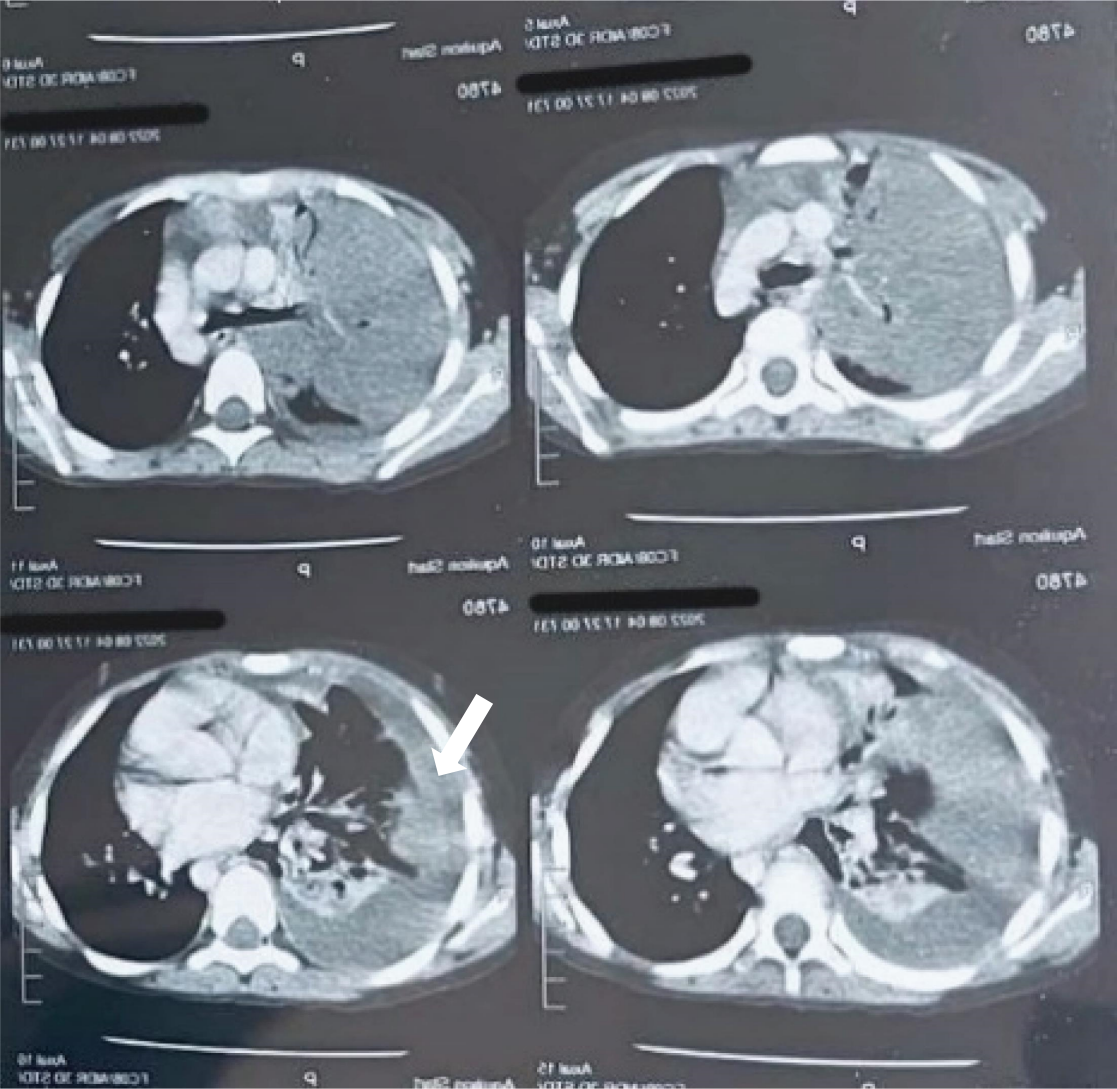
CT chest sagittal plane showing right‐sided empyema (white arrow).

On Day 2, the USG chest revealed right upper and middle lobe consolidation with moderate effusion with multiple septations suggesting empyema thoracis. CT chest was done which showed right upper lobar pneumonia, few hilar mediastinal lymphadenopathies, and right moderate to gross pleural effusion (Figures 2 and 3). On the same day, she was shifted to PICU. Surgical consultation was done and a chest tube was inserted under IVA. Antibiotics were upgraded to intravenous piperacillin/tazobactam and vancomycin.

Streptokinase 2.5‐lakh unit was instilled for 5 days in the pleural cavity in order to dissolve the septation and clear out fluid pockets.

**Figure 4:**
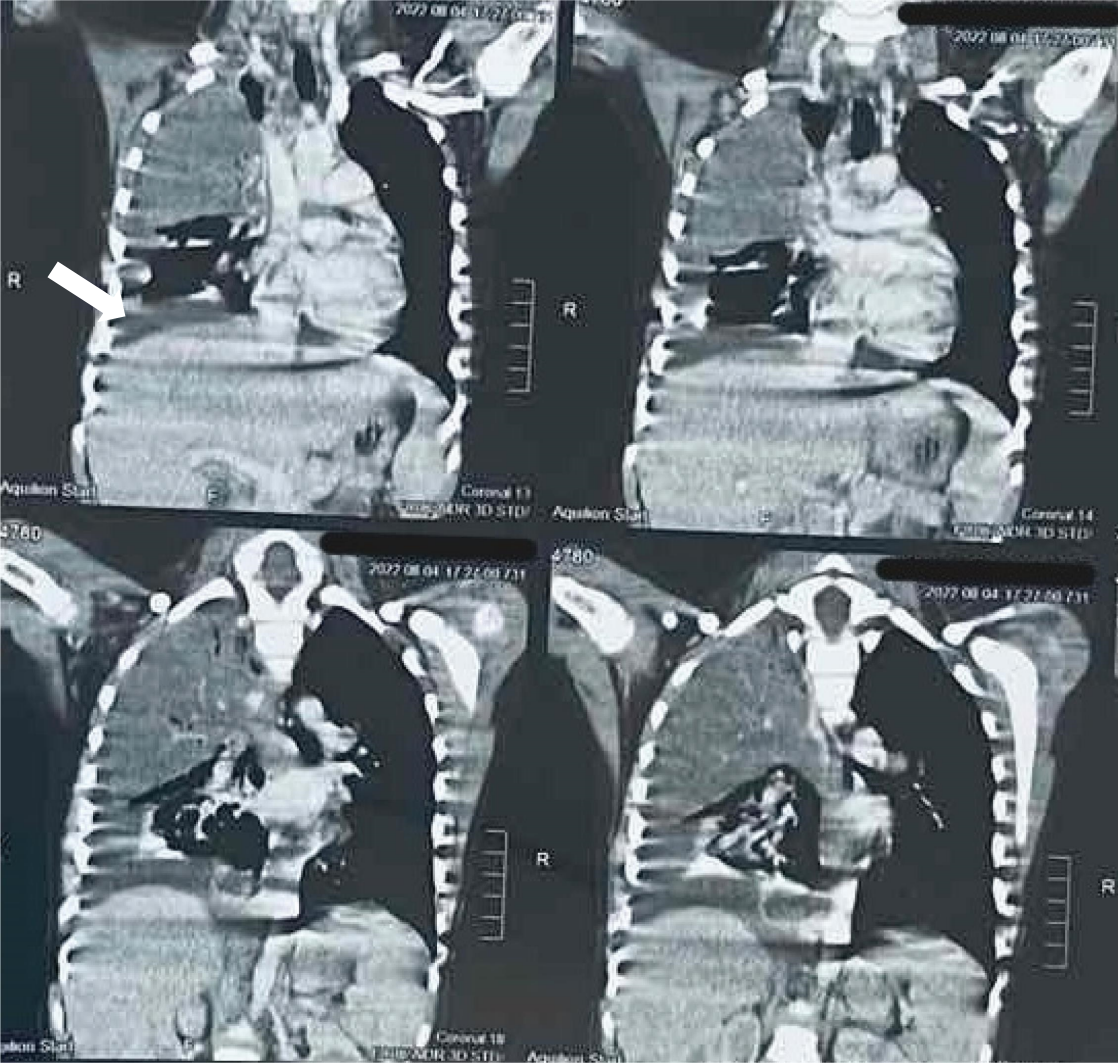
CT chest coronal plane showing right‐sided empyema (white arrow).

On the 4th day of chest tube insertion, the child developed a pulmonary air leak with pneumothorax which was demonstrated in chest x‐ray (Figure 4). There was no trauma during the chest tube insertion procedure, and the child was doing fine for 3 days after the procedure. Pneumothorax is most likely due to erosion of the visceral pleura and necrosis of the superficial lung tissue due to pyogenic infection. Pneumothorax did not subside as expected through the continuous placement of the chest tube drain for a few additional days. Cardiothoracic and vascular surgical consultation was done and was advised for minimal negative pressure suction. Continuous negative suction was applied for 7 days as per the advice.

After 6 days of PICU care, she was shifted to the ward where the IV antibiotics were continued for up to 18 days. Blood culture, urine culture, and sputum culture showed no growth. The pleural fluid drained was seropurulent with an elevated LDH level of 2100 IU/L, but no organisms were found on the culture (Table 1).

**Table 1:** Detailed investigation reports.

Tests	Values	Normal range	Unit
Hemoglobin (Hb)	10.3	12–15.5	g/dL
Total Leucocyte count (TLC)	**9900**	4000–12,000	/mm^3^
Neutrophils (N)	82	40–75	%
Lymphocytes (L)	17	20–45	%
Platelet count (PLT)	260,000	150,000–400,000	/mm^3^
C‐reactive protein (CRP)	**48**	< 6	mg/L
Random blood sugar (RBS)	72	60–125	mg/dL
Sodium (Na)/potassium (K)	135/3.4	135–145/3.5–5	meq/L
Urea (U)/creatinine (Cr)	20/0.5	5–17/0.3–0.7	mg/dL
COVID Ag	Negative		
Serum ADA	178	< 30	IU/L
Serum LDH	716	240–480	IU/L
Mantoux test	5	< 10	mm
Sputum GeneXpert	Negative		
Arterial blood gas (ABG)	pH (7.49) pCO2 (28.2) HCO3(24.1) PO2(58.7)	7.35–7.45, 35–45, 22–28 60–110	mmHg mmol/L mmHg
**Pleural fluid analysis**			
Count	3800		/mm^3^
Neutrophil	2		%
Lymphocytes	98		%
Sugar	200		mg/dL
Protein	3.3		g/dL
LDH	2100		IU/L

**Figure 5:**
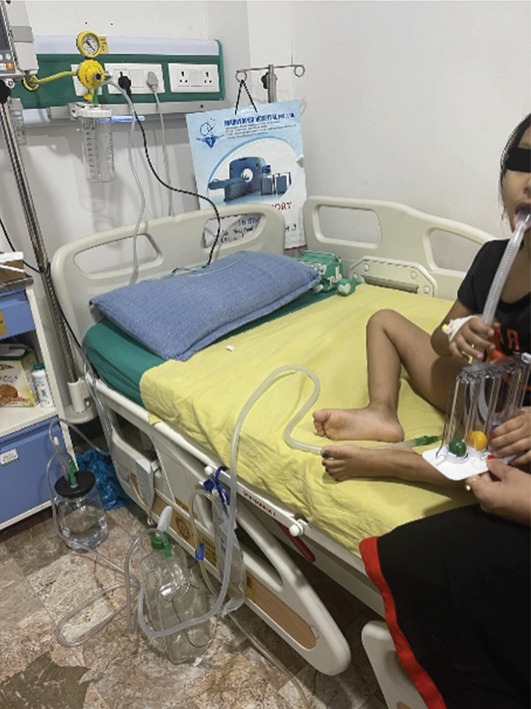
Negative suction being applied to the child.

The child was stable and mobile with chest tube in situ, but the air leak persisted despite continuous negative suctioning. On Day 20, the chest tube was changed and she was discharged home with oral antibiotics (amoxicillin/clavulanic acid and linezolid for 10 more days) and a chest tube in situ as advised by CTVS consultation (Figure 5). The patient′s guardians were taught to change the chest drain bottle under strict aseptic conditions once a day at home. Sterile gloves and water were recommended while cleaning the drain bottle and the parents were trained several times at hospital to change the water until they were confident. Child was also taught to use tri‐ball breathing exerciser to enhance lung inflation. Parents were counselled about the emergency situation that can arise due to sudden dysfunction of the chest tube or the water seal system. They were asked to seek medical help immediately in case of sudden shortness of breath or chest tightness and watch out for chest tube dislodgement or the failure of the water seal system.

**Figure 6:**
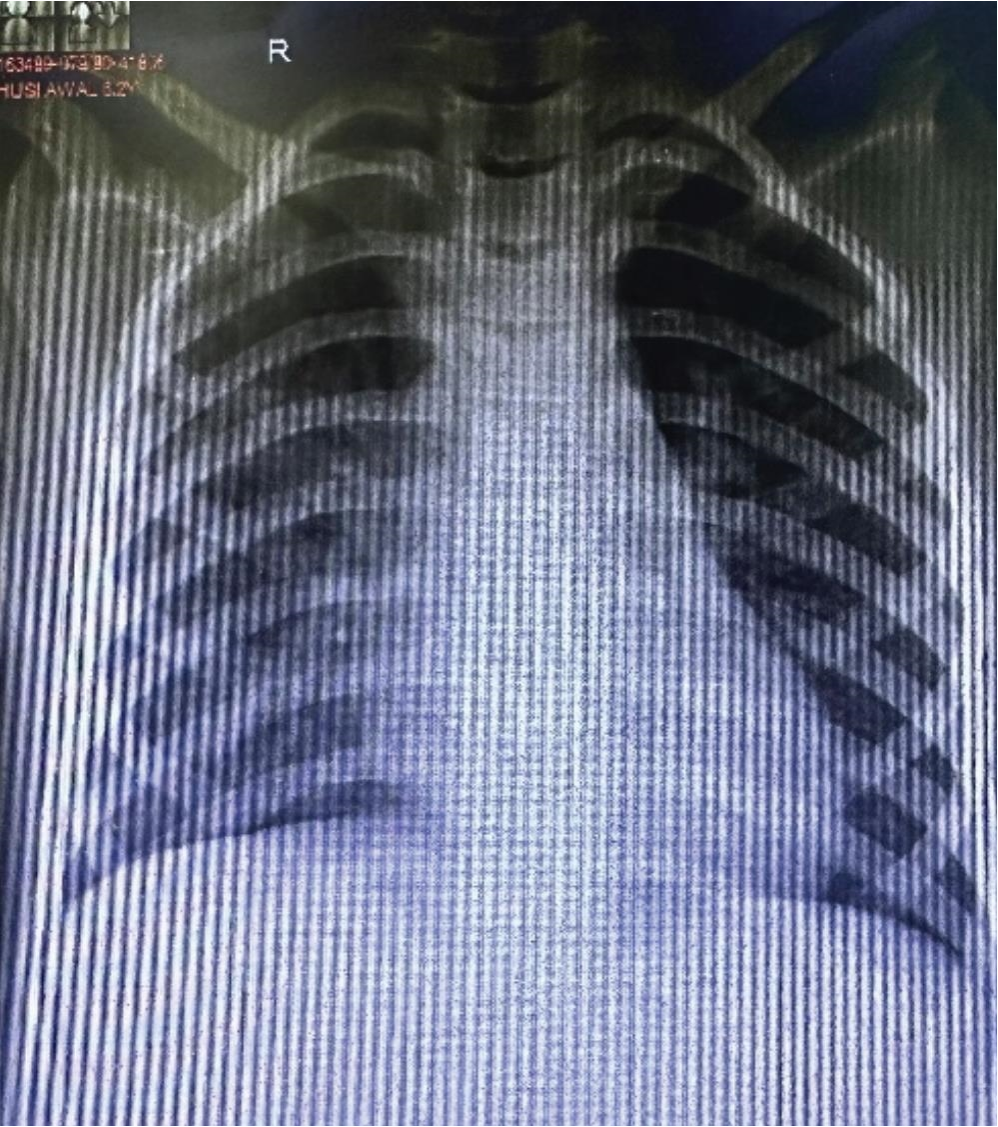
After resolution of air leak.

The child was followed up on regular interval in the hospital and was monitored by serial x‐rays on the days of visit. Initially, there was no improvement in the first visit after a week of home‐based rehabilitation, but subsequent x‐rays showed gradual improvement over a period. After 2 weeks of follow‐up, there was no evidence of pneumothorax in the x‐ray film and there was no air leak in the chest drain bottle. Hence, the chest tube was clamped for 3 h, and chest x‐ray was repeated to confirm the improvement (Figure 6). Then, the chest tube drain was removed, and the child was sent home. The child did well without recurrence of pneumothorax and the recovery was uneventful.

## 3. Discussion

Managing a child with prolonged pneumothorax and air leak complicated by pneumonia poses significant challenges, particularly in light of the escalating issue of drug‐resistant bacteria and the limited efficacy of antibiotics in penetrating the pleural cavity with organized collections. Amidst these complexities, there remains a scarcity of case reports or studies documenting the management of such persistent air leak in children through domiciliary care [4, 8, 9]. Most prolonged air leaks undergo operative management, which may lead to further complications and morbidity [10, 11]. Although devices have been developed for ambulatory pneumothorax management, their effectiveness requires further substantiation through rigorous study. Ambulatory pneumothorax devices are also expensive and not easily available. Prolong chest drain in chest cavity is itself a source of infection. This case represents our inaugural encounter with home‐based management of a prolonged pneumothorax that did not spontaneously resolve despite meticulous hospital care.

One of the crucial learning points from this experience is the recognition of the potential risk of pneumothorax in children with empyema [12]. This underscores the importance of vigilance and comprehensive management strategies in pediatric patients presenting with respiratory infections. As the landscape of bacterial resistance evolves, it becomes imperative for healthcare providers to continually adapt and innovate in their approach to managing complex respiratory conditions in pediatric populations. Though various studies confer the duration of progression to empyema from effusion to be more than a week, few of our cases progressed rapidly to empyema within a few days of admission.

The management of persistent air leak complicated by pneumonia necessitates a multidisciplinary approach, integrating expertise from pediatric pulmonology, infectious diseases, critical care, and thoracic surgery. This collaborative effort is essential for navigating the intricate interplay between bacterial resistance patterns, antibiotic therapy, and the unique anatomical and physiological considerations in pediatric patients and management of chest drain. Furthermore, the incorporation of domiciliary care introduces a novel dimension. This requires meticulous training of the parents with no medical exposure before and monitoring and guiding them via online video and ensuring they adhere to treatment protocols.

Home‐based management should be continuously monitored and follow‐up protocol should be strict in order to avoid mishaps. One should be in continuous touch with the treating physician and any untoward events must be communicated promptly. Exact duration of homebased management is not documented anywhere. So, surgical management options should be discussed with the parents if the air leak is not improving as expected, as this itself might increase the risk of infection and deterioration.

## 4. Conclusion

Despite the challenges posed by persistent air leak in children with pneumonia, there exists a paucity of comprehensive guidelines and evidence‐based recommendations for its management. This highlights the need for further research endeavors, encompassing prospective studies and clinical trials, to elucidate optimal treatment algorithms and refine therapeutic strategies tailored to the pediatric population.

## Funding

No funding was received for this manuscript.

## Consent

Written consent was taken from the parents for publication of this report.

## Conflicts of Interest

The authors declare no conflicts of interest.

## Data Availability

The data that support the findings of this study are available from the corresponding author upon reasonable request.
